# AnkPlex: algorithmic structure for refinement of near-native ankyrin-protein docking

**DOI:** 10.1186/s12859-017-1628-6

**Published:** 2017-04-19

**Authors:** Tanchanok Wisitponchai, Watshara Shoombuatong, Vannajan Sanghiran Lee, Kuntida Kitidee, Chatchai Tayapiwatana

**Affiliations:** 1 Division of Clinical Immunology, Department of Medical Technology, Faculty of Associated Medical Sciences, Chiang Mai University, Chiang Mai, 50200 Thailand; 2 Center of Biomolecular Therapy and Diagnostic, Faculty of Associated Medical Sciences, Chiang Mai University, Chiang Mai, 50200 Thailand; 3 Center of Data Mining and Biomedical Informatics, Faculty of Medical Technology, Mahidol University, Bangkok, 10700 Thailand; 4 Thailand Center of Excellence in Physics, Commission on Higher Education, Bangkok, 10400 Thailand; 5 Department of Chemistry, Faculty of Science, University of Malaya, Kuala Lumpur, 50603 Malaysia; 6 Center for Research and Innovation, Faculty of Medical Technology, Mahidol University, Bangkok, 10700 Thailand

**Keywords:** Ankyrin-protein complexes, Near-native docking pose, Machine learning methods, Decision tree, Logistic regression model, AnkPlex

## Abstract

**Background:**

Computational analysis of protein-protein interaction provided the crucial information to increase the binding affinity without a change in basic conformation. Several docking programs were used to predict the near-native poses of the protein-protein complex in 10 top-rankings. The universal criteria for discriminating the near-native pose are not available since there are several classes of recognition protein. Currently, the explicit criteria for identifying the near-native pose of ankyrin-protein complexes (APKs) have not been reported yet.

**Results:**

In this study, we established an ensemble computational model for discriminating the near-native docking pose of APKs named “AnkPlex”. A dataset of APKs was generated from seven X-ray APKs, which consisted of 3 internal domains, using the reliable docking tool ZDOCK. The dataset was composed of 669 and 44,334 near-native and non-near-native poses, respectively, and it was used to generate eleven informative features. Subsequently, a re-scoring rank was generated by AnkPlex using a combination of a decision tree algorithm and logistic regression. AnkPlex achieved superior efficiency with ≥1 near-native complexes in the 10 top-rankings for nine X-ray complexes compared to ZDOCK, which only obtained six X-ray complexes. In addition, feature analysis demonstrated that the van der Waals feature was the dominant near-native pose out of the potential ankyrin-protein docking poses.

**Conclusion:**

The AnkPlex model achieved a success at predicting near-native docking poses and led to the discovery of informative characteristics that could further improve our understanding of the ankyrin-protein complex. Our computational study could be useful for predicting the near-native poses of binding proteins and desired targets, especially for ankyrin-protein complexes. The AnkPlex web server is freely accessible at http://ankplex.ams.cmu.ac.th.

**Electronic supplementary material:**

The online version of this article (doi:10.1186/s12859-017-1628-6) contains supplementary material, which is available to authorized users.

## Background

Generally, antibodies have several applications in therapies and diagnostics due to the fact that they can be designed to have high affinity with a targeted protein [1, 2]. Due to the complications involved in generating specific antibodies and their large size, alternative scaffolds have been developed to overcome these limitations. One of those novel scaffolds is comprised of Designed Ankyrin Repeat Proteins (DARPins). These ankyrin-proteins have been used more frequently in medical applications [3–5] because of their stability and high affinity for protein targets [6–8]. Moreover, modification of the residues at the variable part of ankyrin allows for increased binding affinity towards the target protein without changes in the basic protein conformation [9]. The high affinity of ankyrin-proteins could be achieved due to random modifications at variable residues *in vitro* [9] and *in silico* prediction of the residues based on the structure of 3-dimensional (3D) complexes [4, 10]. The 3D protein complexes could be determined by X-ray crystallography or NMR spectroscopy, yet few 3D structures of ankyrin-protein complexes have been reported. Most of the structures were monomeric structures or genomics surveys. Therefore, a computational approach, called protein–protein docking, can be used to generate protein complex structures because there were no available reports on the protein complex.

Protein–protein docking is a well-known method for generating protein–protein complexes (poses) using computational methods. The challenging task of identifying the exact bound state of a pair of proteins must consider the following factors: (i) there are several potential ways that a pair of proteins can interact, (ii) the flexibility of the protein, and (iii) changes in the protein conformation after binding [11]. Currently, several software programs have been developed, such as Gramm-X, DOT, ClusPro, and ZDOCK, that provide a rational complex for a pair of proteins, [12]. The ZDOCK program includes initial-stage docking (ZDOCK algorithm) and refinement methods (RDOCK algorithm). The initial-stage docking is designed for searching all possible docking poses [13, 14]. In the refinement stage, the side chains of the docking poses from the ZDOCK algorithm are minimized [15]. The scoring functions (features) of the docking poses are energy terms, such as pairwise shape complementarity (PSC), desolvation (DE), electrostatics (ELEC), and van der Waals. This program has been demonstrated to be one of the most accurate prediction programs in the Critical Assessment of Predicted Interactions (CAPRI) [16].

ZDOCK has successfully predicted several near-native complexes (poses) of antibody-antigen, enzyme-inhibitor and other pairings *via* assessing the CAPRI criteria in the 10 top-rankings based on the features of ZDock, ZRank, or E_RDock. However, successful predictions do not occur for all cases [17–20]. Moreover, the near-native predictors are not selected from an easy ranking of those features, and manual inspections are often needed as well. Note that manual inspections include cluster, density, favourable contact, charge complementarity, buried hydrophobic residues, and overall agreement with the biological data in the literature. Importantly, all protein–protein cases do not agree with the manual inspections. Similar to other reports, the complex-type-dependent combinatorial scoring function was introduced and indicated that the weights of the scoring function were different between protease-inhibitor, antibody-antigen, and enzyme-inhibitor pairings [21]. Therefore, a complicated strategy has to be adopted for obtaining a near-native complex based on certain types of protein–protein complexes.

The near-native docking pose of Ankyrin-Her2 was successfully predicted using ZDOCK and an extra scoring function [10]. Recently, the universal criteria for obtaining the near-native complex of ankyrin-proteins have not been reported, and there was only a computational method that was applied to identify the repeat number of ankyrin-proteins [22]. According to different types of protein-protein complexes, the ankyrin-protein complex requires an individual strategy. Therefore, we aimed to search for explicit criteria to obtain a near-native pose using a set of features generated from one program to avoid using complicated methods or combining scores from several software programs.

In this study, we made a systematic attempt to develop a computational approach for achieving near-native predictors in 10 top-rankings of ankyrin-protein docking poses, which we named AnkPlex. Moreover, this method was generated for (i) analysing and characterizing ankyrin-protein complexes by using a set of informative features that have potential applications and (ii) establishing a user-friendly web server to obtain the desired results without the need to follow complicated mathematical equations generated by the research scientist. The docking poses of seven X-ray complexes of APKs, which had ankyrins with 3 internal domains, were generated using the reliable docking tool ZDOCK. The construction of the docking poses calculated by PSC alone and summation of PSC + DE + ELEC demonstrated there were different numbers of near-native docking poses. The steps for AnkPlex establishment included (i) balancing the near-native and non-near-native poses; (ii) processing the dataset through machine learning of a decision tree algorithm (DT) and a logistic regression (LG) with a combination of 11 features; (iii) selecting the efficient predictive models of DT and LG; and (iv) processing the dataset by combining models of DT and LG.

## Method

### Datasets

X-ray crystal structures of ankyrin-protein complexes (APKs) were collected from the *Protein Data Bank* (PDB) database for 41 APKs reported up to May 2014. Analyses of the 41 APKs were performed through data pre-processing using the following steps: (i) APKs containing 3-internal-domain were included; (ii) redundant APKs were excluded; (iii) APKs were filter based on the recognition areas [7]; and (iv) alpha, beta, and alpha–beta proteins were selected using the SCOP database [23]. Nine X-ray crystal structures of APKs (called Ank9) were obtained, as summarized in Additional file 1: Table S1. Subsequently, seven of the APKs were randomly selected as training complexes (Ank-TRN), including complex 1 (C_1_), complex 2 (C_2_), complex 3 (C_3_), complex 4 (C_4_), complex 5 (C_5_), complex 6 (C_6_), and complex 7 (C_7_). At the same time, the rest of the APKs, including unknown 1 (U_1_) and unknown 2 (U_2_), were designated the test group (Ank-TEST). In order to avoid the distinct results from the different selections of training and test sets, other 35 possible datasets were constructed and were used to generate the predictive models for the identification of near-native poses.

**Fig. 1:**
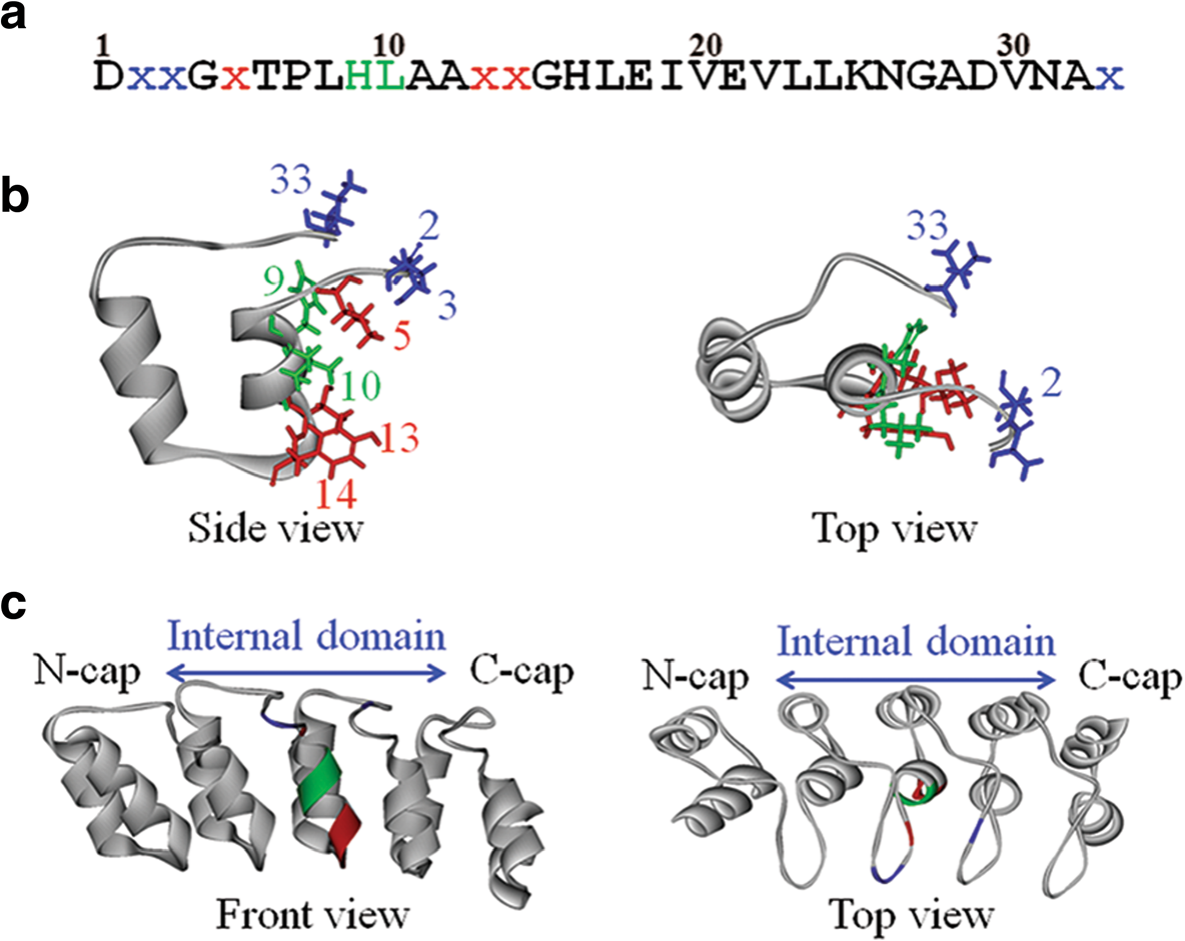
The molecular architecture of ankyrin and its three recognition areas, as shown in ribbon style. **a** Amino acid sequence of an internal repeat [7] in which the recognition residues are shown in three colours. **b** The ribbon style of an internal repeat of ankyrin related to the above sequence. **c** The structure of the 3 internal domains of ankyrin flanked by the N-cap and C-cap. The recognition area consisted of six variable residues [7] (*red* and *blue* are positioned on the helix and turn, respectively) and two constant amino acids (*green*) on the second domain

The docking poses of Ank-TRN and Ank-TEST were regenerated by using the protein docking software ZDOCK [13, 14]. Two versions of the docking poses were generated, which were different in terms of energy calculations (especially PSC) and the combination of PSC, DE, and ELEC (PSC + DE + ELEC). Then, all the generated-docking poses were superimposed with the original X-ray crystal structures and were calculated for the root-mean-square deviation of the Cα atom (Cα-RMSD) value. The docking poses that presented Cα-RMSD values ≤10 Å were designated to be near-native poses or positive samples, whereas the docking poses that presented Cα-RMSD values >10 Å were defined as non-near-native poses or negative samples [24]. The numbers of near-native poses for the two versions of the docking poses were compared. In addition to screening near-native poses by the Cα-RMSD value, eight binding residues of the APKs on the second domain of ankyrin (Fig. 1) were used for filtering near-native poses based on the recognition areas (regKp).

### Feature extraction

Based on observations of the generation of the features, ankyrin-protein docking poses were generated for the energy features using the ZDOCK protocol [13, 14] (a set of 5 features) and the RDOCK protocol [15] (a set of 6 features). Five features, including ZDock, ZRankElec, ZRnakSolv, ZRank, and ZRankVdw, were obtained from the protein-docking protocol (ZDOCK) using the CHARMm force field [25]. At the same time, six features, including E_vdw1, E_elec1, E_vdw2, E_elec2, E_sol, and E_RDock, were calculated from the docking refinement protocol (RDOCK) using the CHARMm polar H force field [25]. The energy equation used in RDOCK was the same as ZDOC. However, the ankyrin-protein docking poses were minimized before calculation. The details of the 11 features (A, B, C, D, E, F, G, H, I, J, K) are described below:

- ZDock (A) is the Pairwise Shape Complementarity (PSC) score and it was optionally augmented with the electrostatics (ELEC) and the desolvation energy (DE). In this study, the *ZDock* score was calculated using the following equation:

1 $$ ZDock\  score=\alpha PSC+ D E+\beta ELEC $$

where *α* and *β* have the default values of 0.01 and 0.06, respectively.

- ZRankElec (B) is the long-range electrostatic energy and the only fully charged side-chain, as represented in the following equation:

2 $$ ZRankElec\left( i, j\right) = 332\frac{q_i{q}_j}{{r^2}_{i j}} $$

where *q*_*i*_ and *q*_*j*_ are the charges on ankyrin and the protein atoms, respectively. The *r*_*ij*_ in the equation stands for the distance between the atoms of ankyrin and the protein.

- ZRankSolv (C) is the desolvation term based on the Atomic Contact Energy (ACE).

3 $$ ZRankSolv\left( i, j\right) = {a_i}_j $$

where *a*_*ij*_ is the ACE score.

- ZRank (D) is a linear combination of ZRankVdw, ZRankElec, and ZRankElec.

4 $$ ZRank\  score= ZRankElec + ZRankSolv + ZRankVdw $$

- ZRankVdw (E) is the van der Waals and short-range electrostatics energy with a distance between the atom pair being less than 5.0 Å. This calculation was based on the parameters of the CHARMm 19 polar hydrogen potential. The *ZRankVdw* score was calculated as follows:

5 $$ ZRankVdw\left( i, j\right) = {\varepsilon_i}_j\left[{\left[\frac{\sigma_{i j}}{r_{i j}}\right]}^{12}-2{\left[\frac{\sigma_{i j}}{r_{i j}}\right]}^6\right] $$

where *ε*_*ij*_ and *σ*_*ij*_ are the depth and the width, respectively, of the coefficient for the CHARMm 19 polar H.

- E_vdw1 (F) and E_vdw2 (H) are the van der Waals energy, as presented in Equation (5), of the 1^st^ and the 2^nd^ minimized structure of the ankyrin-protein docking poses, respectively.
- E_elec1 (G) and E_elec2 (I) are the electrostatic energy, as presented in Equation (2), of the ankyrin-protein docking poses processed for the 1^st^ and the 2^nd^ minimization, respectively.
- E_sol (J) is the desolvation energy, as shown in Equation (4), of the 2^nd^ minimization of the ankyrin-protein docking poses.
- E_RDock (K) is the summation of E_sol and (0.9 × E_elec2).

### Construction of learning method

Several learning models were constructed including decision tree (DT), logistic regression (LG), artificial neural network (ANN), and support vector machine (SVM) using Ank-TRN (C_1_-C_7_). As shown in Additional file A1: Table S5, SVM yielded 100% near-native poses in the internal testing sets but could not obtain any near-native pose in the external testing sets. The DT and ANN provided the near-native poses from both internal and external testing sets. According to a dataset of C_5_, the DT was superior in achieving the near-native poses of internal testing sets than the ANN. The LG provided a weighted summation that could rank the docking poses to achieve the near-native poses in the 10 top-rankings. As a consequence, the DT and the LG were selected to construct an ensemble model.

**Fig. 2:**
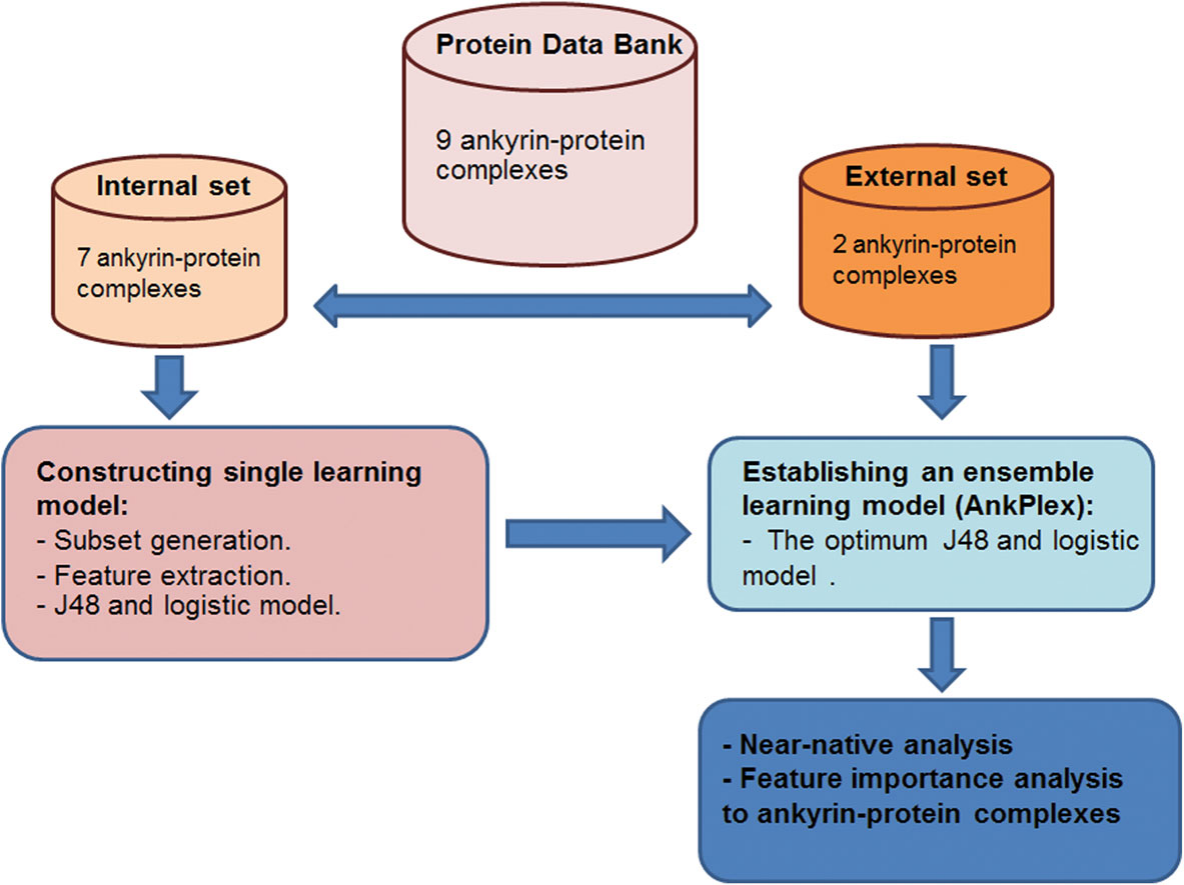
The flowchart system for the proposed AnkPlex

To identify the near-native docking poses of APKs, a learning method named AnkPlex was established by combining a decision tree (DT) and a logistic method (LG). The decision trees and the logistic regression methods were selected due to the fact that they provide a high number of predicted positive values (true near-native poses). The logistic regression especially provided a weighted summation that was finally ranked to search for near-native poses in 10 top-rankings. All 11 features and all datasets were used to build the DT and LG models. The Ank-TRN (7 APKs) and the Ank-TEST (2 APKs) were evaluated by AnkPlex using the following steps, as shown Fig. 2:

1. The number of near-native poses and non-near-native poses were balanced. ZDOCK using PSC + DE + ELEC and regKp provided 699 near-native poses and 44,334 non-near-native poses of Ank-TRN. The non-near-native poses were randomly clustered into 65 groups (≈44,334/669). Therefore, each training set was composed of the same near-native poses and different groups of non-near-native poses.

2. A predictive model using the DT and the LG models was established. All 11 features were combined and generated as feature subsets (i.e., A, B, C, D, E, F, G, H, I, J, K, AB, AC, AD, AE, AF, AG, AH, AI, AJ, AK, BC, BD, …, ABCDEFG). The total number of feature subsets was calculated to be 4,095 by following this equation:

6 $$ L={\displaystyle \sum_{r=1}^{11}\frac{11!}{r!\left(11- r\right)!}}. $$

The DT model was established from 4,095 feature subsets and 65 training sets using the J48 algorithm [26, 27]. The parameters of the DT model were set with the confidence factor, the minimum number of objects, and the number of folds for reduced error pruning of 0.25, 2, and 3, respectively. Additionally, the LG model was constructed from the same feature subsets and training set with a ridge estimator [28] in which the maxis and the ridge were defined as −1 and 1.oE-8, respectively. Subsequently, the learning methods were generated by implementation of the DT and the LG models using the WEKA program [27].

3. An efficient predictive model of the DT and the LG models was selected. Ank-TRN consisted of 7 APKs and was submitted to the learning method for predicting the near-native poses. True positive rates (TPrate) greater than 50% were used as the cut-off value for an efficient learning method. The learning methods that demonstrated a TPrate greater than 50% were selected to further establish an ensemble learning model.

4. Ensemble methods were established. The ensemble learning method, named AnkPlex, was constructed by randomly integrating the DT-based learning models (OLM_DT_) and the LG-based learning models (OLM_LG_) from Step 3 for reducing the number of non-near-native docking poses. The main process of the proposed method, AnkPlex, for increasing the number of TPs (reducing non-near-native poses) consisted of the following steps: (i) only predicted positive samples (PPV_DT_) derived from OLM_DT_ were select, (ii) a logistic score (LGS) on PPV_DT_ using the LG model was calculated, and (iii) PPV_DT_ was ranked according to LGS and the 10 top-ranking poses that demonstrated the highest LGS were selected. The near-native pose(s) or the true positive (TP) in the 10 top-ranking poses were our targets. The summation score of AnkPlex was defined in the equation given as Equation (7) on *C*_*i*_, where *i* = 1, 2, …, 7, and *Y*_*i*_ would be set as 1 in case TP was found in the 10 top-ranking poses. Otherwise, *Y*_*i*_ would be set as 0. Finally, the score of AnkPlex was the summation product, as defined in the following equation:

7 $$ \# P P={\displaystyle \sum_{i=1}^7{Y}_i} $$

where # *PP* belongs to Ank-TRN containing seven complexes (*C*_*1*_ *, C*_*2*_ *, …, C*_*7*_) and *Y*_*i*_ would be set as 1 when TP appears in the 10 top-ranking poses. Otherwise, *Y*_*i*_ would be set as 0. The number of #PP_TRN_ indicated the sample of Ank-TRN in which the LGS score was among the 10 top-ranking near-native poses. The number of #PP_TEST_ showed the LGS score of the Ank-TEST was among the 10 top-ranking poses.

### Validation

The prediction performance of the AnkPlex method was evaluated by using 10-fold cross-validation (10-fold CV). The method validation parameters, including accuracy (ACC), sensitivity (SEN), and precision (PRES), were calculated using the following equations:

8 $$ \mathrm{Accuracy} = \kern0.5em \frac{TP+ TN}{\left( TP+ TN+ FP+ FN\right)}\times 100 $$

9 $$ \mathrm{Sensitivity} = \kern0.5em \frac{TP}{\left( TP+ FN\right)}\times 100 $$

10 $$ \mathrm{Precision} = \kern0.5em \frac{TP}{\left( TP+ FP\right)}\times 100 $$

where TP, TN, FP, and FN are the numbers of true positive, true negative, false positive, and false negative results, respectively.

## Results and discussion

### Analysis of ankyrin-protein docking dataset

There were a few ankyrin-protein complexes (APKs) reported in the PDB database. Forty-one ankyrin complexes, with the number of internal domains ranging from 2–7, have been reported (up to May, 2014). The highest number, 19 complexes, of ankyrin-proteins contained 3 internal domains. Furthermore, the APKs that reacted with the target using recognition areas were selected. Focusing on target proteins, only proteins with common folding structures, i.e., alpha-, beta- and alpha-beta structures, were considered. Therefore, nine complexes, which included 1SVX, 4ATZ, 3Q9N, 1AWC, 2BKK, 2Y1L, 4DRX, 2P2C, and 4HNA, were used in this study. These nine complexes were randomly divided into two groups, i.e., 7 complexes as Ank-TRN and 2 complexes as Ank-TEST.

To optimize the ZDOCK calculation, X-ray crystal structures of APK-TRN, including seven APKs, were calculated with different feature calculations, including the PSC and the PSC + DE + ELEC. The total number of docking poses, including near-native and non-near-native poses, was 54,000 poses (54Kp). Subsequently, the numbers of near-native poses calculated by PSC and PSC + DE + ELEC were compared. As shown in Table 1, the average number of near-native poses calculated by PSC + DE + ELEC (116.57 ± 51.05) was twice as high as the number calculated using PSC (63.29 ± 41.43). To increase the predictive accuracy, binding sites on the second domain of Ank-TRN defined by Bintz et al. [7] were used for filtering near-native poses based on the recognition areas (regKp). The number of regKp calculated by PSC + DE + ELEC was observed to be slightly reduced (95.57 ± 52.58). According to the ZDOCK program suggestion, the near-native poses were identified in the top 2,000 poses (2Kp) ranked by the ZRank feature [13, 14]. The 2Kp were selected from the total docking poses of regKp compared to the near-native poses from regKp. The near-native poses of 2Kp (47.14 ± 31.49) substantially decreased two-fold compared to regKp. Thus, it can be concluded that 2Kp ranked by the ZRank feature was not suitable for screening near-native poses because of the exclusion of some near-native poses. Interestingly, screening by regKp resulted in a high number of near-native poses and an extremely reduced number of non-near-native poses. The results suggested that the ZDOCK calculation using PSC + DE + ELEC and the screening based on the recognition areas (regKp) were the optimal calculations because this procedure was capable of incorporating near-native poses and eliminating non-near-native poses. However, the number of non-near-native poses generated with regKp still remained high, which indicated that an alternative learning method is necessary for ruling out non-near-native poses.

**Table 1:** Number of docking poses classified as near-native and non-near-native in Ank-TRN (C_1_-C_7_) and Ank-TEST (U_1_ and U_2_)

Complex	Near-native				Non-near-native	
	PSC^a^	PSC + DE + ELEC^b^			PSC + DE + ELEC^b^	
	54Kp	54Kp	regKp	2Kp	54Kp	regKp
C_1_	81	157	136	83	53,843	6,626
C_2_	4	72	53	19	53,921	4,865
C_3_	125	194	179	54	53,806	7,055
C_4_	83	131	104	74	53,869	4,450
C_5_	31	101	59	8	53,891	8,565
C_6_	84	42	28	18	53,958	6,177
C_7_	35	119	110	74	53,881	6,596
Total	443	816	669	330	384,169	44,334
Mean	63.29	116.57	95.57	47.14	53,881	6,333.43
std.	41.43	51.05	52.58	31.49	49.74	1,377.39
U_1_	ND^c^	83	83	57	ND^c^	6,833
U_2_	ND^c^	183	32	13	ND^c^	7,183

^a^ZDOCK was calculated by PSC alone. ^b^ZDOCK was calculated by combining PSC, DE, and ELEC. ^c^The data were not used for analysis

### Establishing learning methods

According to the ZDOCK calculations of Ank-TRN, 11 features of near-native poses and non-near-native poses were generated. Univariate statistical approaches were employed to perform exploratory data analysis using average and standard deviations for summarizing important patterns. As shown in Table 2, five features that were generated by the ZDOCK protocol demonstrated the significant differences between the near-native poses and the non-near-native poses with a *p-value <*0.001. As presented in Table 2, the five top-ranked features included E_RDock (−11.96 ± 9.40/1.72 ± 12.10), ZRankElec (8.22 ± 16.56/29.17 ± 22.41), ZRank (−54.03 ± 25.84/−21.82 ± 31.33), E_elec2 (−18.21 ± 8.85/−8.18 ± 10.25), and E_sol (4.43 ± 6.26/9.07 ± 8.87). Almost all the features that were calculated using the RDOCK protocol were significantly different, except E_elec1 (*p* = 0.178) and E_vdw2 (*p* = 0.010). Subsequently, 11 features calculated with the ZDOCK calculation were applied to establish the learning methods.

**Table 2:** Summary of statistical analysis of near-native and non-near-native poses of ankyrin-target complexes

Feature	Near-native	Non-near-native	*p*-value
ZDock	36.73 ± 6.32	33.41 ± 4.87	<0.001
ZRankElec	8.22 ± 16.56	29.17 ± 22.41	<0.001
ZRank	−54.03 ± 25.84	−21.82 ± 31.33	<0.001
ZRankSolv	3.66 ± 6.99	7.88 ± 10.10	<0.001
ZRankVdw	−65.91 ± 20.44	−58.87 ± 20.98	<0.001
E_vdw1	−56.21 ± 57.81	−47.46 ± 101.79	<0.001
E_elec1	-1.05 ± 2.18	-0.94 ± 2.37	0.18
E_vdw2	−70.01 ± 42.72	−74.33 ± 43.00	0.01
E_elec2	−18.21 ± 8.85	−8.18 ± 10.25	<0.001
E_sol	4.43 ± 6.26	9.07 ± 8.87	<0.001
E_RDock	−11.96 ± 9.40	1.72 ± 12.10	<0.001

Eleven features of each of the near-native poses (669) and non-near-native poses (44,334) calculated from Ank-TRN based on the recognition areas (regKp) were used to establish the learning methods. Based on the unbalanced number of docking poses, training sets were generated by clustering the non-near-native poses and the near-native poses into 65 sets (44,334/669). Eleven features were calculated from each training set and were ordered to generate 4,095 feature sets. The DT-based learning models (OLM_DT_) and the LG-based learning models (OLM_LG_) were established using the 4,095 feature sets. The learning methods demonstrated the average of the true positive rate to be greater than 50% (TPrate ≥ 50%), and consisted of 4,762 OLM_DT_ and 2,688 OLM_LG_. The learning models that represented TPrate ≥ 50% with the 10 top-ranking poses of %ACC are shown in Table 3 (10 top-rankings of OLM_DT_) and Table 4 (10 top-rankings of OLM_LG_). As a result, ABDEHIJK_g14 of OLM_DT_ exhibited the highest %ACC with %TPrate ≥ 50%. This learning method consisted of sequential combination feature sets that included ZDock (A), ZRankElec (B), ZRank (D), ZRankVdw (E), E_vdw2 (H), E_elec2 (I), E_sol (J), and E_RDock (K) calculated from non-near-native dataset number 13. In addition, CDFGJ_g10 of OLM_LG_ also demonstrated the highest %ACC with %TPrate ≥ 50%. The percentage of precision (%PRES) for all the 10 top-ranking poses of OLM_DT_ and OLM_LG_ was low, which indicated that there was a high number of false positive results (FP). To diminish the number of FP, only the 10 top-ranking poses based on the ZRank score were selected to represent the true positive poses (TP). If the TP were found in 10 top-ranking poses from each Ank-TRN, #PP was designated 1. Thus, the #PP-values of seven Ank-TRN (#PP_TRN_) were in the range of 0 to 7. As shown in Tables 3 and 4, the maximum values of the #PP_TRN_ of OLM_DT_ and OLM_LG_ were only 6. Therefore, the individual learning method of OLM_DT_ or OLM_LG_ was not capable of providing the maximum value for #PP_TRN_.

**Table 3:** Comparison of performances of 10 top-ranking OLM_DT_ among various types of features and datasets in terms of 10-fold cross-validation

Rank	OLM_DT_	PRES(%)	REC(%)	ACC(%)	#PP_TRN_
1	ABDEHIJK_g14	82.10 ± 8.49	70.58 ± 15.03	6.96 ± 4.82	6
2	ADEFHIK_g25	81.64 ± 9.08	72.05 ± 14.65	7.05 ± 5.06	6
3	ABDEGHIJK_g14	81.14 ± 8.83	73.66 ± 16.61	6.83 ± 4.68	6
4	BCEGHIJK_g13	81.04 ± 8.37	72.88 ± 14.37	6.03 ± 3.31	6
5	AEFGHJK_g36	81.03 ± 8.71	73.85 ± 13.05	6.91 ± 5.17	6
6	ABFIJK_g16	80.84 ± 8.48	69.17 ± 14.68	6.33 ± 4.32	6
7	ABDEFGIJK_g25	80.84 ± 7.98	75.54 ± 8.88	6.41 ± 3.80	6
8	ABIJK_g16	80.82 ± 8.43	69.17 ± 14.68	6.31 ± 4.31	6
9	ABCGHJK_g13	80.80 ± 7.86	73.55 ± 12.44	6.30 ± 3.98	6
10	ABDEFGIK_g25	80.78 ± 8.09	75.67 ± 9.12	6.38 ± 3.73	6

11 feathers (A, B, C, D, E, F, G, H, I, J, K) are ZDock, ZRankElec, ZRank, ZRankSolv, ZRankVdw, E_vdw1, E_elec1, E_vdw2, E_elec2, E_sol, E_RDock

**Table 4:** Comparison of performances of 10 top-ranking OLM_LG_ among various Types of features and datasets in terms of 10-fold cross-validation

Rank	OLM_LG_	PRES(%)	REC(%)	ACC(%)	#PP_TRN_
1	CDFGJ_g10	74.76 ± 11.62	67.83 ± 14.80	4.91 ± 3.75	6
2	CDFJ_g10	74.75 ± 11.59	67.66 ± 15.08	4.90 ± 3.75	6
3	CDJ_g10	74.74 ± 11.55	67.83 ± 14.80	4.91 ± 3.76	6
4	CDGJ_g10	74.72 ± 11.55	67.83 ± 14.80	4.91 ± 3.76	6
5	BCDEFGJ_g10	74.64 ± 10.30	68.06 ± 17.77	4.47 ± 2.86	4
6	BCDEGHJ_g10	74.60 ± 10.41	68.27 ± 17.78	4.48 ± 2.86	4
7	BCDEFGHJ_g10	74.54 ± 10.17	68.45 ± 17.85	4.46 ± 2.85	4
8	BCDEFHJ_g10	74.51 ± 10.20	68.69 ± 17.58	4.47 ± 2.84	4
9	ACDGJ_g36	74.48 ± 11.91	68.96 ± 15.52	4.94 ± 3.80	5
10	BCDEGJ_g41	74.48 ± 10.78	67.93 ± 16.77	4.57 ± 3.21	4

11 feathers (A, B, C, D, E, F, G, H, I, J, K) are ZDock, ZRankElec, ZRank, ZRankSolv, ZRankVdw, E_vdw1, E_elec1, E_vdw2, E_elec2, E_sol, E_RDock

### Ensemble learning method to generate AnkPlex

**Fig. 3:**
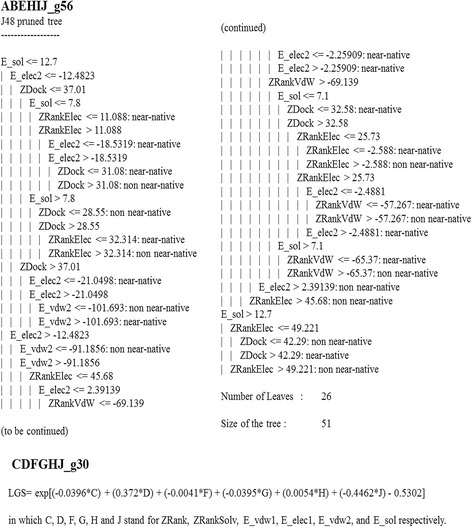
Characteristics of the optimal AnkPlex

To enhance the prediction efficacy of the generated learning methods, 4,762 of the DT-based learning models (OLM_DT_) and 2,688 of the LG-based learning models (OLM_LG_) were randomly combined to generate an ensemble model. Interestingly, the combination of the ensemble model from ABEHIJ_g56 of OLM_DT_ and CDFGHJ_g30 of OLM_LG_ demonstrated superior prediction efficiency due to the fact that this ensemble model (ABEHIJ_g56- CDFGHJ_g30) achieved maximum values for #PP_TRN_ and #PP_TEST_ of 7 and 2, respectively. Therefore, the ensemble model, ABEHIJ_g56- CDFGHJ_g30, was designated to be an ensemble computational model for predicting the near-native docking pose of APKs or “AnkPlex” (Fig. 3). To compare the prediction efficiency of the ensemble model, AnkPlex with the single learning models, the total number of TP and the first TP of each Ank-TRN were used for the evaluation. As shown in Table 5, the single learning models of OLM_DT_ (ABEHIJ_g56) and OLM_LG_ (CDFGHJ_g30) provided a #PP_TRN_ value of 6. The first TP of C_5_ predicted by ABEHIJ_g56 and the C_6_ predicted by CDFGHJ_g30 were found at pose numbers 14 and 19. This result indicated that a single learning model could not produce all the true positive poses. In the case of the Ank-TEST, OLM_DT_ could not provide the value for the #PP_TEST_, whereas the #PP_TEST_ of OLM_LG_ was comparable to AnkPlex. Consequently, it can be concluded that the ensemble model, AnkPlex, was capable of including a #PP_TRN_ value of 7 and a #PP_TEST_ value of 2, which suggested that the prediction efficacy of AnkPlex was superior to the single learning model. In addition, the predictive models generated from other 35 possible datasets demonstrated the average number of #PP_TRN_ and #PP_TEST_ value of 6.78 ± 0.42 and 2 ± 0.00, respectively. This indicated that different selections of training and test sets had no effect in the generation of the learning models for predicting the near-native poses.

**Table 5:** Comparison of performances of AnkPlex with single learning method and ZDOCK program^a^

Method	Number of TP docking poses (rank)								
	C_1_	C_2_	C_3_	C_4_	C_5_	C_6_	C_7_	U_1_	U_2_
2Kp(ZDOCK)	0 (13)	1 (10)	6 (1)	7 (1)	1 (10)	1 (10)	8 (1)	0 (14)	0 (104)
OLM_DT_ (ABEHIJ_g56)	1 (4)	1 (4)	8 (1)	7 (1)	0 (14)	2 (1)	7 (1)	0 (13)	0 (160)
OLM_LG_ (CDFGHJ_g30)	2 (1)	1 (3)	8 (1)	10 (1)	1 (9)	0 (19)	7 (1)	1 (6)	1 (5)
AnkPlex	2 (1)	1 (3)	8 (1)	10 (1)	1 (9)	3 (2)	7 (1)	1 (6)	1 (5)

^a^The number of TP docking poses is the summation of the TP docking poses found in 10 top-ranking poses, where the maximum and the minimum are 10 and 0, respectively. The rank is denoted by the order in which the first TP docking poses are found. For example, on C_6_, AnkPlex yields three TP docking poses on the top 10 ranking poses, and the orders of the three TP docking poses are 2, 8, and 9. Thus, the rank of AnkPlex on C_6_ is 2

According to the ZDOCK program recommendations, near-native docking poses could be found in 2Kp, as indicated by a high ZDock score, low E_RDock, or low ZRank [15, 17–20]. Particularly, the ZRank score provided a #PP_TRN_ value of 6, which was higher compared to the values for other features (Table 6). Thus, 2Kp ranked by the ZRank score was selected to identify #PP. As shown in Table 5, the #PP_TRN_ and the #PP_TEST_ of 2Kp could not reach the maximum value. In addition, the first TP of 2Kp was found to be a lower order number compared to AnkPlex. These results indicated that ZRANK was able to identify the most accurate near-native poses. Nevertheless, it would not be applied for all cases. Thus, the combined feature, AnkPlex, could be used to adjust this solution.

**Table 6:** Number of near-native poses in 10 top-ranking poses obtained from ZDOCK program with 2Kp

Feature	Number of near-native poses in 10 top-ranking poses										
	C_1_	C_2_	C_3_	C_4_	C_5_	C_6_	C_7_	#PP_TRN_	U_1_	U_2_	#PP_TEST_
ZDock	0	0	0	3	0	0	4	2	0	0	0
ZRankElec	2	0	0	0	0	0	0	1	0	0	0
ZRank	0	1	6	7	1	1	8	6	0	0	0
ZRankSolv	0	0	0	7	1	0	0	2	0	0	0
ZRankVdw	0	0	4	0	0	3	1	3	0	0	0
E_vdw1	0	0	0	0	0	0	0	0	0	0	0
E_elec1	0	0	0	0	0	7	0	1	0	0	0
E_vdw2	0	0	0	0	0	0	0	0	0	0	0
E_elec2	0	0	0	0	0	6	0	1	0	0	0
E_sol	0	0	0	9	1	0	0	2	0	0	0
E_RDock	8	0	0	4	0	0	5	3	0	0	0

To apply the AnkPlex for investigating the ankyrin-protein complex, Ank^GAG^1D4 was used to study this learning model. Ank^GAG^1D4 is an artificial ankyrin that contains 3 internal domains and was designed as an antiretroviral agent. Ank^GAG^1D4 was able to bind to the N-terminal domain of the capsid protein (CA^NTD^) of HIV-1 [3]. Recently, the X-ray structure Ank^GAG^1D4 was already constructed. However, the complex structure of Ank^GAG^1D4-CA^NTD^ was not detected [4]. Thus, we generated the docking poses of Ank^GAG^1D4-CA^NTD^ and performed re-scoring with AnkPlex. The results revealed that three near-native structures of Ank^GAG^1D4-CA^NTD^ were found in the 10 top-rankings. The recognition residues of Ank^GAG^1D4-CA^NTD^ interactions were further investigated by observing interacting distances ≤ 5 Å. As a result, one docking pose showed that residue R18 was located on the recognition areas of CA^NTD^ and two docking poses demonstrated residues R132 and R143 played key roles in the interaction with Ank^GAG^1D4 (data not shown). This result correlated with previous ELISA results. A point mutation of R18A on helix 1 and R132A and R143A on helix 7 of CA^NTD^ showed negative binding to Ank^GAG^1D4. Thus, R18, R132 and R143 were the key residues of CA^NTD^ binding to Ank^GAG^1D4 [4]. According to computational analysis of Ank^GAG^1D4 using this learning model, AnkPlex could not only discriminate the near native docking poses of Ank^GAG^1D4-CA^NTD^ complex but also demonstrated the correct orientation of the recognition area to CA^NTD^.

### Feature importance analysis

**Fig. 4:**
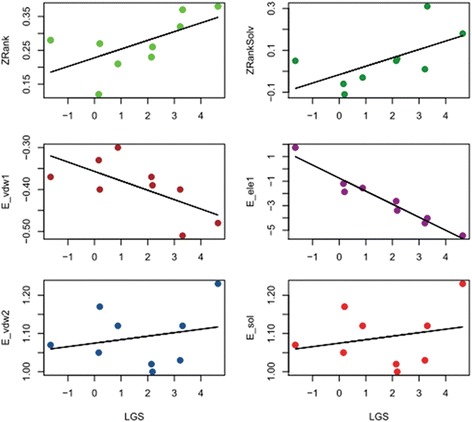
The correlation coefficients between the dot products of the features and their weights of the best-ranking LGS for the near-native poses in the nine ankyrin-protein complexes

Identification of informative features among the 11 features was critical for designing a powerful learning model and for understanding and obtaining insights into the ankyrin-protein docking poses. Based on the six features (CDFGHJ) used in the calculation of the LGS in AnkPlex, the Pearson correlation coefficients (*R* values) were used to identify the correlation between LGS and the weights of the six features to obtain the near-native poses. As shown in Fig. 4 and Additional file 1: Table S3, the three top-ranked *R* values of the six features consisted of ZRank (*R* = 0.60), ZRankSolv (*R* = −0.56), and E_sol (*R* = 0.54), which indicated that these three features played an important role in the AnkPlex model for distinguishing near-native poses.

The ZRank score was ranked as the 1^st^ informative feature according to the highest *R* values (0.60). The characteristics of the ZRank score between the near-native and the non-near-native poses were significantly different, with *p* < 0.001, as shown in Table 2. To confirm the important roles of the ZRank score in AnkPlex, the ensemble learning method based on AnkPlex was constructed without ZRank (C). As a result, as demonstrated in Additional file 1: Table S2, the AnkPlex lacking ZRank (OLM_DT_(ABEHIJ_g56)–OLM_LG_(DFGHJ_g30) was able to obtain #PP_TRN_ = 1 and #PP_TEST_ = 0. However, AnkPlex (OLM_DT_(ABEHIJ_g56)–OLM_LG_(DFGHJ_g30) achieved success with #PP_TRN_ = 7 and #PP_TEST_ = 2. Therefore, ZRank was concluded to be an important feature of AnkPlex due to the fact that it could enhance the predictive performance of near-native poses.

Since ZRank is a linear combination of van der Waals (ZRankVDW), electrostatics (ZRankElec), and desolvation energy (ZRankSolv), one of them had to be identified as the most important. From Additional file 1: Table S3, it is evident that the ZRankVDW (van der Waals interaction) was more dominant than the ZRankElec and the ZRankSolv. Recently, ZRank was developed by correcting the weight of the energies and combining a pairwise interface potential in which the weight of van der Waals was higher than the original ZRank [29]. This result supports the theory that van der Waals is an important property for near-native docking poses of ankyrin-protein pairings.

ZRankSolv and E_sol were the desolvation energies estimated by the summation of the Atomic Contact Energy (ACE) in which the difference between the two features was in the force field calculation and side chain orientation. ZRankSolv and E_sol were the 2^nd^ and the 3^rd^ informative features with *R* values of −0.56 and 0.54, respectively. Moreover, these two features showed differences in their characteristics between near-native and non-near-native poses, with *p* < 0.001, as shown in Table 2. Our experimental results (see Additional file 1: Table S2) demonstrated that AnkPlex lacked ZRankSolv (D), i.e., OLM_DT_(ABEHIJ_g56)–OLM_LG_(CFGHJ_g30) provided #PP_TRN_ = 6 and #PP_TEST_ = 1. Similar to E_sol, the performance of AnkPlex lacked E_sol, i.e., (OLM_DT_(ABEHIJ_g56)–OLM_LG_(CDFGH_g30)) yielded #PP_TRN_ = 6 and #PP_TEST_ = 1. This result indicated that the absence of ZRankSolv and E_sol slightly reduced the predictive performance of AnkPlex. However, these two features were required for predicting near-native poses. ZRankSolv was a component of ZRank. This outcome emphasized that desolvation was important for obtaining near-native poses in the ankyrin-protein interaction. Additionally, it was also required for the accuracy of other protein–protein complexes [30–32].

The LGS score was a combination of the energy determined in the interaction area between ankyrin and proteins of ≤5 Å. In AnkPlex, the interaction area on ankyrin was located on variable and conserved residues of the L-shaped repeat belonging to the internal repeats, the N-terminal repeat and the C-terminal repeat [7]. The functional variable residues on ankyrin were required for the recognition of the target protein by using the available solvent-accessible surface [7, 33, 34]. To observe the variable area used for calculating the energy, analysis of the first TP of the near-native of Ank9 was carried out to count the variable and conserved residues in the interaction area. The result, which was presented in Additional file 1: Table S4, showed that there was 43.75 ± 12.90% of the interaction area on ankyrin belonging to the variable residues, and 58.50 ± 11.36% of this area represented the hydrophobic residues. This result indicated that the interaction energy was calculated on both the variable and the conserved residues. Therefore, computing the energy term at the interface of the variable residues could provide a score to distinguish between the near-native and the non-near-native docking poses. As a consequence, the calculation for evaluating the score based on the desired area could be applied in the docking algorithm.

According to the hydrophobicity on the interface in AnkPlex (see Additional file 1: Table S4), the interactions between ankyrin and proteins were comprised of 18.05 ± 6.32%, hydrophobic–hydrophobic, 43.25 ± 13.70%, hydrophobic–hydrophilic and 38.70 ± 4.48%. hydrophilic–hydrophilic interactions. Moreover, the percentage of hydrophobic–hydrophobic interactions in the non-near-native pose was observed to be reduced by 12.69 ± 7.31%, as shown in Additional file 1: Table S4. However, the percentage of hydrophobic–hydrophilic interactions in the near-native pose increased to 50.32 ± 8.89%. This outcome indicated that the recognition site on ankyrin for the target protein was adopted to have hydrophobic and hydrophilic interactions, which promoted the solvent-accessible property [35]. Because LGS is modified from atom-based potential without considering the type of the hydrophobicity scale, the high LGS of the non-near-native docking pose could be calculated from the hydrophobic–hydrophilic interaction instead of the hydrophobic–hydrophobic interaction.

## Conclusions

An ensemble method, named AnkPlex, was constructed for fast prediction of near-native states of ankyrin-protein complexes. The AnkPlex model was constructed based on a combination of features generated from the ZDOCK program without using manual inspections. AnkPlex successfully obtained the near-native poses of nine ankyrin-protein complexes in the 10 top-ranking poses. ZRank, which is a combination of electrostatic, desolvation, and van der Waals energy, was the most important feature in AnkPlex. In addition, van der Waals was the dominant feature for obtaining the near-native docking poses. To develop the method for predicting near-native poses of protein complexes, we have implemented easy access to the best models for the scientific community on a web server. AnkPlex (http://ankplex.ams.cmu.ac.th) is freely available online.

## Additional files

Additional file 1: Table S1. Informative characteristics of nine ankyrin-protein complexes. **Table S2.** Comparison of performances of 10 top-ranking among ensemble learning models^a^. **Table S3.** Features values of the best-ranking LGS for the near-native poses in the nine ankyrin-protein complexes. **Table S4.** Types of interaction pair and hydrophobic residues on interface (≤5 Å) of the nine ankyrin-protein complexes. **Table S5.** The percentage of predictable true near-native poses of the internal and external testing sets on leaning methods of decision tree (DT), logistic regression (LG), artificial neural network (ANN), and support vector machine (SVM). (DOC 125 kb)

Additional file 2: Supplementary datasets used in this study. (XLS 13646 kb)

## Abbreviations

ACE: Atomic Contact Energy
APKs: ankyrin-protein complexes
Cα-RMSD: Carbon alpha root-mean-square deviation
DARPins: Designed Ankyrin Repeat Proteins
DE: desolvation
DT: decision tree
ELEC: electrostatics
LG: logistic model
LGS: logistic score
PPV: predicted positive value
PSC: shape complementarity
