# Supplementary material for: AnkPlex: algorithmic structure for refinement of near-native ankyrin-protein docking
Source: BMC Bioinformatics. 2017 Apr 19;18:220. doi: 10.1186/s12859-017-1628-6 (PMC5395911; doi:10.1186/s12859-017-1628-6)
Supplement: Supplementary file 1 — Informative characteristics of nine ankyrin-protein complexes. Table S2. Comparison of performances of 10 top-ranking among ensemble learning modelsa. Table S3. Features values of the best-ranking LGS for the near-native poses in the nine ankyrin-protein complexes. Table S4. Types of interaction pair and hydrophobic residues on interface (≤5 Å) of the nine ankyrin-protein complexes. Table S5. The percentage of predictable true near-native poses of the internal and external testing sets on leaning methods of decision tree (DT), logistic regression (LG), artificial neural network (ANN), and support vector machine (SVM). (DOC 125 kb) [file 12859_2017_1628_MOESM1_ESM.doc]

**Table S1** Informative characteristics of nine ankyrin-protein complexes

|  |  | **Ankyrin** |  | **Target protein** | |
| --- | --- | --- | --- | --- | --- |
|  | **PDB id** | **Length (residues)** |  | **Length (residues)** | **Protein family defined by SCOP** |
| C_1_^a^ | 1SVX | 169 |  | 395 | alpha and beta proteins (a/b) |
| C_2_ ^a^ | 4ATZ | 154 |  | 201 | all beta proteins |
| C_3_ ^a^ | 3Q9N | 158 |  | 141 | alpha and beta proteins (a/b) |
| C_4_ ^a^ | 1AWC | 153 |  | 110 | all alpha proteins |
| C_5_ ^a^ | 2BKK | 169 |  | 264 | alpha and beta proteins (a+b) |
| C_6_ ^a^ | 2Y1L | 169 |  | 159 | alpha and beta proteins (a/b) |
| C_7_ ^a^ | 4DRX | 169 |  | 437 | alpha and beta proteins (a/b) |
| U_1_ ^b^ | 2P2C | 158 |  | 98 | alpha and beta proteins (a/b) |
| U_2_ ^b^ | 4HNA | 159 |  | 431 | alpha and beta proteins (a/b) |

^a^Ankyrin training complexes (Ank-TRN) were composed of complex 1 (C_1_), complex 2 (C_2_), complex 3 (C_3_), complex 4 (C_4_), complex 5 (C_5_), complex 6 (C_6_), and complex 7 (C_7_).

^b^Ankyrin testing complexes (Ank-TEST) comprised , unknown 1 (U_1_) and unknown 2 (U_2_).

**Table S2** Comparison of performances of 10 top-ranking among ensemble learning models^a^.

| **Ensemble learning models**  **(OLM_DT_- OLM_LG_)** | **#PP_TRN_** | **#PP_TEST_** |
| --- | --- | --- |
| A_g56 - A_g30 | 3 | 0 |
| B_g56 - B_g30 | 1 | 0 |
| C_g56 - C_g30 | 6 | 0 |
| D_g56 - D_g30 | 2 | 0 |
| E_g56 - E_g30 | 2 | 0 |
| F_g56 - F_g30 | 3 | 0 |
| G_g56 - G_g30 | 1 | 0 |
| H_g56 - H_g30 | 1 | 1 |
| I_g56 - I_g30 | 1 | 0 |
| J_g56 - J_g30 | 2 | 0 |
| K_g56 - K_g30 | 4 | 0 |
| ABEHIJ_g56 - DFGHJ_g30 | 1 | 0 |
| ABEHIJ_g56 - CFGHJ_g30 | 6 | 1 |
| ABEHIJ_g56 - CDGHJ_g30 | 7 | 1 |
| ABEHIJ_g56 - CDFHJ_g30 | 7 | 2 |
| ABEHIJ_g56 - CDFGJ_g30 | 7 | 1 |
| ABEHIJ_g56 - CDFGH_g30 | 6 | 1 |

^a^The ensemble model having #PP_TRN_ =7 and #PP_TEST_ =2 is the more powerful model for predicting the ankyrin-protein complex

**Table S3** Features values of the best-ranking LGS for the near-native poses in the nine ankyrin-protein complexes

|  | **ZRankElec** | **ZRankVdw** | **ZRank** | **ZRankSolv** | **E_vdw1** | **E_elec1** | **E_vdw2** | **E_sol** |
| --- | --- | --- | --- | --- | --- | --- | --- | --- |
| C_1_ | −24.61 | −73.13 | −90.10 | 7.64 | −65.96 | −1.54 | −66.07 | 6.70 |
| C_2_ | 9.11 | −90.38 | −85.25 | −3.99 | −76.54 | −4.86 | −78.35 | −2.60 |
| C_3_ | −17.44 | −126.24 | −138.98 | 4.70 | −88.76 | −0.77 | −98.22 | 7.40 |
| C_4_ | −17.41 | −92.71 | −114.49 | −4.37 | −74.12 | −3.98 | −75.65 | −3.80 |
| C_5_ | 16.17 | −96.13 | −77.44 | 2.52 | −77.88 | 1.77 | −86.74 | 2.40 |
| C_6_ | 14.38 | −132.13 | −106.15 | 11.60 | −95.24 | −8.60 | −101.17 | 12.10 |
| C_7_ | −6.38 | −114.91 | −121.66 | −0.37 | −36.24 | −4.22 | −86.10 | 0.60 |
| U_1_ | −6.16 | −98.53 | −94.46 | 10.22 | −75.32 | 0.03 | −73.77 | 9.50 |
| U_2_ | −11.60 | −63.50 | −69.60 | 5.51 | 859.67 | −2.74 | 857.59 | 6.10 |

**Table S4** Types of interaction pair and hydrophobic residues on interface (≤5Å) of the nine ankyrin-protein complexes.

|  | **Best-ranking of near-native^a^ (%)** | | | | | | | **Best-ranking of non-near-native^b^ (%)** | | | | | |
| --- | --- | --- | --- | --- | --- | --- | --- | --- | --- | --- | --- | --- | --- |
|  | **Interaction pair** | | |  | **Ankyrin** |  |  | | **Interaction pair** | | |  | **Ankyrin** |
|  | **pho-pho^c^** | **pho-phi^d^** | **phi-phi^e^** |  | **pho^d^** |  |  | | **pho-pho^c^** | **pho-phi^d^** | **phi-phi^e^** |  | **pho^d^** |
| C_1_ | 5.30 | 74.93 | 19.77 |  | 79.80 |  |  | | 9.18 | 64.63 | 26.20 |  | 70.94 |
| C_2_ | 15.53 | 50.19 | 34.27 |  | 51.60 |  |  | | 19.08 | 43.35 | 37.56 |  | 51.81 |
| C_3_ | 16.31 | 42.72 | 40.97 |  | 48.37 |  |  | | 14.47 | 46.29 | 39.25 |  | 50.69 |
| C_4_ | 29.09 | 27.08 | 43.83 |  | 42.23 |  |  | | 8.02 | 35.25 | 56.73 |  | 32.48 |
| C_5_ | 20.76 | 40.98 | 38.26 |  | 34.89 |  |  | | 4.56 | 44.59 | 50.85 |  | 39.60 |
| C_6_ | 17.13 | 32.11 | 50.76 |  | 40.50 |  |  | | 23.57 | 56.86 | 19.57 |  | 51.83 |
| C_7_ | 19.62 | 37.25 | 43.13 |  | 52.29 |  |  | | 12.46 | 52.33 | 35.21 |  | 62.95 |
| U_1_ | 16.93 | 45.18 | 37.89 |  | 57.42 |  |  | | 2.44 | 57.56 | 40.00 |  | 39.29 |
| U_2_ | 21.77 | 38.82 | 39.42 |  | 42.23 |  |  | | 20.39 | 52.04 | 27.57 |  | 60.58 |
| ave. | 18.05 | 43.25 | 38.70 |  | 49.93 |  |  | | 12.69 | 50.32 | 36.99 |  | 51.13 |
| std. | 6.32 | 13.70 | 8.48 |  | 13.19 |  |  | | 7.31 | 8.89 | 11.77 |  | 12.47 |

^a^The highest LGS of near-native docking poses. ^b^The highest LGS of non-near-native docking poses. ^c^hydrophobic–hydrophobic interaction between ankyrin and target protein. ^d^hydrophobic–hydrophilic interaction between ankyrin and target protein. ^e^hydrophilic–hydrophilic interaction between ankyrin and target protein. ^f^hydrophobic residue on anyrin.

**Table S5** The percentage of predictable true near-native poses of the internal and external testing sets on leaning methods of decision tree (DT), logistic regression (LG), artificial neural network (ANN), and support vector machine (SVM)

| **Methods** | **Training set** | **Testing set (%)** | | | | | | |
| --- | --- | --- | --- | --- | --- | --- | --- | --- |
|  |  | **C_1_** | **C_2_** | **C_3_** | **C_4_** | **C_5_** | **C_6_** | **C_7_** |
| **DT** | **C_1_** | 60.29 | 0.00 | 0.00 | 0.00 | 0.00 | 0.00 | 0.74 |
|  | **C_2_** | 0.00 | 33.96 | 0.00 | 22.64 | 0.00 | 0.00 | 0.00 |
|  | **C_3_** | 16.20 | 0.00 | 33.52 | 8.94 | 0.00 | 0.00 | 9.50 |
|  | **C_4_** | 0.00 | 0.00 | 13.46 | 74.04 | 0.00 | 0.00 | 12.50 |
|  | **C_5_** | 0.00 | 0.00 | 0.00 | 3.39 | 23.73 | 0.00 | 0.00 |
|  | **C_6_** | 7.14 | 0.00 | 0.00 | 0.00 | 0.00 | 78.57 | 0.00 |
|  | **C_7_** | 6.36 | 0.00 | 12.73 | 34.55 | 0.00 | 4.55 | 41.82 |
| **LG** | **C_1_** | 16.18 | 0.00 | 0.00 | 2.21 | 0.00 | 0.00 | 0.00 |
|  | **C_2_** | 0.00 | 0.00 | 0.00 | 5.66 | 0.00 | 0.00 | 0.00 |
|  | **C_3_** | 55.87 | 0.00 | 19.55 | 20.11 | 0.00 | 0.00 | 6.70 |
|  | **C_4_** | 66.35 | 0.00 | 13.46 | 71.15 | 1.92 | 0.00 | 22.12 |
|  | **C_5_** | 1.69 | 0.00 | 0.00 | 5.08 | 0.00 | 0.00 | 0.00 |
|  | **C_6_** | 28.57 | 0.00 | 0.00 | 7.14 | 0.00 | 60.71 | 7.14 |
|  | **C_7_** | 52.73 | 0.00 | 9.09 | 50.91 | 1.82 | 0.91 | 12.73 |
| **ANN** | **C_1_** | 63.24 | 0.00 | 0.00 | 1.47 | 0.00 | 0.00 | 0.00 |
|  | **C_2_** | 0.00 | 32.08 | 0.00 | 3.77 | 0.00 | 0.00 | 0.00 |
|  | **C_3_** | 40.78 | 0.00 | 34.64 | 15.08 | 0.00 | 0.56 | 16.76 |
|  | **C_4_** | 4.81 | 0.00 | 16.35 | 75.96 | 0.00 | 0.00 | 34.62 |
|  | **C_5_** | 0.00 | 0.00 | 0.00 | 0.00 | 3.39 | 0.00 | 0.00 |
|  | **C_6_** | 7.14 | 0.00 | 7.14 | 0.00 | 0.00 | 71.43 | 10.71 |
|  | **C_7_** | 23.64 | 0.00 | 17.27 | 41.82 | 0.00 | 1.82 | 54.55 |
| **SVM** | **C_1_** | 100.00 | 0.00 | 0.00 | 0.00 | 0.00 | 0.00 | 0.00 |
|  | **C_2_** | 0.00 | 100.00 | 0.00 | 0.00 | 0.00 | 0.00 | 0.00 |
|  | **C_3_** | 0.00 | 0.00 | 100.00 | 0.00 | 0.00 | 0.00 | 0.00 |
|  | **C_4_** | 0.00 | 0.00 | 0.00 | 100.00 | 0.00 | 0.00 | 0.00 |
|  | **C_5_** | 0.00 | 0.00 | 0.00 | 0.00 | 100.00 | 0.00 | 0.00 |
|  | **C_6_** | 0.00 | 0.00 | 0.00 | 0.00 | 0.00 | 100.00 | 0.00 |
|  | **C_7_** | 0.00 | 0.00 | 0.00 | 0.00 | 0.00 | 0.00 | 100.00 |
